# COPD significantly increases cerebral and cardiovascular events in hypertensives

**DOI:** 10.1038/s41598-021-86963-z

**Published:** 2021-04-12

**Authors:** Maria Perticone, Raffaele Maio, Benedetto Caroleo, Edoardo Suraci, Salvatore Corrao, Giorgio Sesti, Francesco Perticone

**Affiliations:** 1 Department of Medical and Surgical Sciences, University Magna Græcia, Viale Europa–Loc. Germaneto, 88100 Catanzaro, Italy; 2 Azienda Ospedaliero-Universitaria Mater Domini, Catanzaro, Italy; 3 Department of Internal Medicine 2, National Relevance and High Specialization Hospital Trust, University of Palermo, Palermo, Italy; 4 Department of Clinical and Molecular Medicine, University La Sapienza of Rome, Rome, Italy

**Keywords:** Cardiology, Medical research, Risk factors

## Abstract

Essential hypertension and chronic obstructive pulmonary disease often coexist in the same patient. The aim of this study was to evaluate whether the addition of chronic obstructive pulmonary disease modifies the risk of cardiovascular events in hypertensives. We enrolled 1728 hypertensives. Study outcomes included fatal and non-fatal cardiovascular stroke and myocardial infarction, and cardiovascular death. During a mean follow-up of 57 months there were 205 major adverse cardiovascular events (2.47 per 100 pts/yr): cardiac (n117; 1.41 per 100 pts/yr) and cerebrovascular (n = 77; 0.93 per 100 pts/yr). In hypertensives with chronic obstructive pulmonary disease we observed a greater number of cardiovascular events than in hypertensives without respiratory disease (133 [5.55 per 100 pts/yr) vs 72 [1.22 per 100 pts/yr], respectively. The addition of chronic obstructive pulmonary disease to hypertension increased the incidence of total and non-fatal stroke of more than nine- (2.42 vs 0.32 per 100 pts/yr) and 11-fold (2.09 vs 0.22 per 100 pts/yr), respectively. The same trend was observed for total (2.88 vs 0.81 per 100 pts/yr) and non-fatal (2.67 vs 0.79 per 100 pts/y) myocardial infarction. The presence of chronic obstructive pulmonary disease in hypertensives significantly increases the risk of stroke, myocardial infarction and major adverse cardiovascular events.

## Introduction

Life expectancy has improved dramatically over recent decades^1^. The ageing of populations and the increase of non-communicable diseases lead to a rapid rise of the number of people with multiple health conditions^2^. Of interest, this multimorbidity, defined as the coexistence of two or more chronic conditions in the same individual, has a relevant impact on clinical practice, disease prognosis and national healthcare systems costs^3^.

Essential hypertension (HT) and chronic obstructive pulmonary disease (COPD) are two of the most prevalent chronic diseases worldwide and are responsible for more than 8 and 3 million deaths per year respectively^4,5^, representing two of the leading causes of death in industrialized Countries. Of interest, both conditions are often present in the same patient, probably because they share some common risk factors^6^, contributing to the increase of the cardiovascular (CV) risk burden in a multiplicative manner^7–10^.

Even if it is well known that HT represents the most frequent comorbidity in COPD, occurring in more than 50% of patients^11^, few data exist on the prevalence of respiratory disease in hypertensive patients and on its role in the appearance of clinical outcomes^5^, probably because COPD still remains an underdiagnosed chronic disease despite its great impact on CV morbidity and mortality. Consequently, the prognostic significance of COPD in hypertensive patients, especially with regard to stroke occurrence, needs to be further elucidated. Thus, the aim of the present study was to evaluate the role of COPD in the appearance of fatal and non-fatal stroke and myocardial infarction (MI), and CV death, in a large and well-characterized cohort of hypertensive patients.

## Results

### Study population

In Table 1 we reported the baseline demographic, clinical and biochemical characteristics of the whole study population and of the two groups, divided according to the presence/absence of COPD, separately. The mean age of the whole study population was 61 ± 12 years; there were 1046 males (60.5%) and 1161 (67.2%) habitual smokers; systolic blood pressure (SBP) and diastolic blood pressure (DBP) values were 143 ± 17 and 89 ± 11 mmHg, respectively. Patients with both HT and COPD, representing 30.3% of the whole study population, were older with a higher prevalence of males and smokers. In addition, they had a higher body mass index (BMI), SBP, pulse pressure (PP) and hs-CRP, and lower values of estimated glomerular filtration rate (e-GFR) and HDL cholesterol. No significant differences were observed in anti-hypertensive treatment between groups, except for a reduced use of β-blockers in HT + COPD patients (Table 2), as expected. Similarly, no significant differences between groups (68 vs 67% in the HT and HT + COPD patients, respectively) were observed in the percentage of patients reaching blood pressure (BP) target.

**Table 1:** Baseline demographic, hemodynamic, and biochemical characteristics of the whole study population and of the two groups separately.

Variables	ALL (n = 1728)	HT (n = 1204)	HT + COPD (n = 524)	P
Gender, M/F	1046/682	678/526	368/156	0.0001
Age, years	61 ± 12	59 ± 11	68 ± 11	0.0001
Smoke, yes/no	1161/567	734/469	427/98	0.0001
BMI, kg/m^2^	26.7 ± 3.3	26.6 ± 2.6	27.1 ± 4.5	0.009
SBP, mmHg	143 ± 17	142 ± 17	146 ± 17	0.0001
DBP, mmHg	89 ± 11	93 ± 11	87 ± 10	0.003
PP, mmHg	58 ± 15	56 ± 14	62 ± 16	0.0001
e-GFR, ml/min/1.73m^2^	100 ± 31	109 ± 32	81 ± 17	0.0001
hs-CRP, mg/l	4.0 ± 3.4	3.6 ± 3.3	5.1 ± 3.4	0.0001
Total cholesterol, mg/dl	184 ± 28	185 ± 30	183 ± 22	0.101
HDL-cholesterol, mg/dl	50 ± 14	51 ± 14	48 ± 13	0.0001
LDL-cholesterol, mg/dl	108 ± 28	108 ± 30	108 ± 23	0.937
Triglycerides, mg/dl	129 ± 64	128 ± 69	130 ± 53	0.710

BMI: body mass index; DBP: diastolic blood pressure; e-GFR: estimated glomerular filtration rate; HDL: high density lipoprotein; hs-CRP: high sensitivity C reactive protein; LDL: low density lipoprotein; PP: pulse pressure; SBP: systolic blood pressure.

**Table 2:** Baseline pharmacological treatment in the whole study population and in the two groups separately.

Drugs	ALL (n = 1728)	HT (n = 1204)	HT + COPD (n = 524)	P
ACE-i/ARBs, n (%)	699 (77)	497 (77)	202 (77)	0.313
CCBs, n (%)	285 (31)	199 (31)	86 (32)	0.991
β-blockers, n (%)	227 (25)	174 (27)	53 (20)	0.018
α-blockers, n (%)	37 (4)	27 (4)	10 (4)	0.795
Diuretics, n (%)	377 (41)	265 (41)	112 (43)	0.817
Associations, n (%)	517 (57)	370 (57)	147 (56)	0.289
BP target	1178 (68)	825 (68)	353 (67)	0.676
Aspirin, n (%)	380 (22)	269 (22)	121 (23)	0.780
LAMA, n (%)			285 (54)	
LABA, n (%)			160 (30)	
LAMA/LABA, n (%))			79 (15)	

ACE-i: angiotensin converting enzyme inhibitors; ARBs: angiotensin receptor inhibitors; BP: blood pressure; CCBs: calcium channel blockers; LABA: long-acting β_2_-agonists; LAMA: long-acting muscarinic antagonists.

### Survival analysis

During the follow-up period (57 ± 27 months, range 13–168), 205 patients experienced a new major adverse cardiovascular event (MACE) (2.47 per 100 pts/yr) (Table 3): 117 patients (57%; 1.41 per 100 pts/yr) had coronary events, while 77 patients (37%; 0.93 per 100 pts/yr) experienced cerebrovascular events; 11 patients (5.0%; 0.13 per 100 pts/yr) had CV death (6 deaths were attributable to arrhythmic causes, 5 to pulmonary embolism, and 1 to aortic rupture). In addition, we observed 35 non-CV deaths: 18 (0.30 per 100 pts/yr) occurred in HT and 17 (0.71 per 100 pts/yr) in COPD groups, respectively (P = 0.029).

**Table 3:** Incidence of study outcomes during the follow-up period in the whole study population and in the two groups separately.

Outcomes	All	HT	HT + COPD	P
**MACE, n (%)**	205 (11.9)	72 (5.9)	133 (25.3)	0.0001
Incidence, n per 100 pts/yr	2.47	1.22	5.55	
**Myocardial infarction**				
Total, n (%)	117 (6.8)	48 (3.9)	69 (13.1)	0.0001
Incidence, n per 100 pts/yr	1.41	0.81	2.88	
Non-fatal (%)	111 (6.5)	47 (3.9)	64 (12.2)	0.0001
Incidence, n per 100 pts/yr	1.34	0.79	2.67	
**Stroke**				
Total, n (%)	77 (4.4)	19 (1.5)	58 (11.0)	0.0001
Incidence, n per 100 pts/yr	0.93	0.32	2.42	
Non-fatal, n (%)	63 (3.6)	13 (1.0)	50 (9.5)	0.0001
n per 100 pts/yr	0.76	0.22	2.09	

MACE: major adverse cardiovascular events.

**Figure 1:**
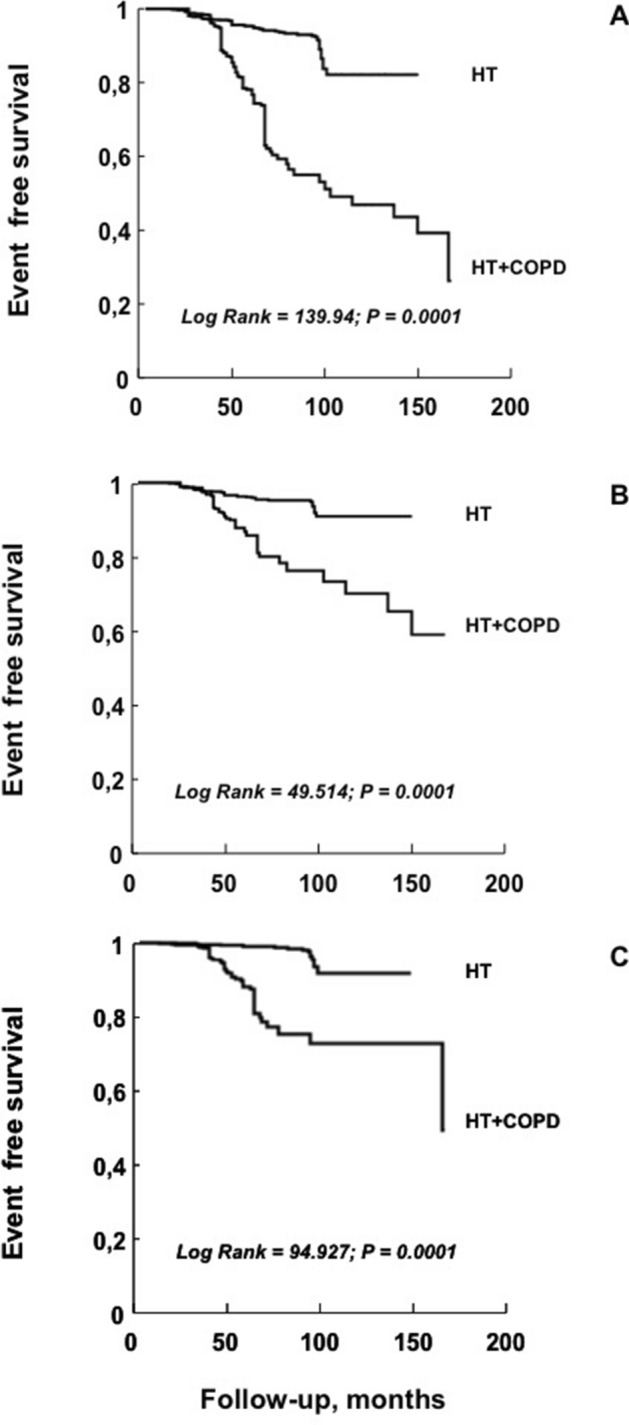
Kaplan–Meier curves for MACE (**A**), total myocardial infarction (**B**) and total stroke (**C**) in hypertensive patients with and without COPD.

In comparison with HT patients, those in the HT + COPD group had significantly higher CV events; in particular, we observed 133 (25.3%; 5.55 per 100 pts/y) vs 72 (5.9%; 1.22 per 100 pts/y) MACE, respectively. The same trend was observed for all the other study outcomes (Table 3). Notably, HT + COPD patients, in comparison with HT group, exhibited an incidence of more than three-fold higher for both total (2.88 vs 0.81 per 100 pts/yr) and non-fatal (2.67 vs 0.79 per 100 pts/y) myocardial infarction (MI), respectively. Clinically relevant, the addition of COPD in hypertensive patients significantly increased the incidence of total and non-fatal stroke of more than nine- (2.42 vs 0.32 per 100 pts/yr) and 11-fold (2.09 vs 0.22 per 100 pts/yr), respectively. In Fig. 1 we graphically reported the Kaplan–Meier curves for MACE and both total MI and stroke.

### Cox regression analysis

At multiple Cox regression analysis, COPD resulted as the main and independent predictor of the incident risk of the all study outcomes in the whole study population. In addition, hs-CRP turned out to be an independent predictor for MACE and total and non-fatal stroke, while e-GFR was retained in the model as independent predictor for total and non-fatal MI (Table 4). Notably, and clinically relevant, the presence of COPD is able to considerably increase the risk of both total (HR = 9.053; 95% CI 4.067–17.792) and non-fatal (HR = 10.847; 95% CI 5.091–23.113) stroke, to a greater extent than observed for total (HR = 1.920; 95% CI 1.223–3.013) and non-fatal (HR = 1.881; 95% CI 1.187–2.982) MI (Table 4).

**Table 4:** Multiple Cox regression analysis for study outcomes.

Outcomes	Hazard ratio	95%CI	P
**MACE (n = 205)**			
COPD	3.290	2.276–4.756	0.0001
hs-CRP	1.050	1.009–1.093	0.017
**Total stroke (n = 77)**			
COPD	9.119	4.631–17.958	0.0001
hs-CRP	1.075	1.010–1.145	0.024
**Non fatal stroke (n = 63)**			
COPD	10.908	5.108–23.295	0.0001
hs-CRP	1.082	1.010–1.160	0.026
**MI (n = 117)**			
COPD	1.928	1.228–3.030	0.004
e-GFR	0.864	0.787–0.949	0.002
**Non fatal MI (n = 111)**			
COPD	1.886	1.189–2.992	0.007
e-GFR	0.871	0.792–0.957	0.004

Data are adjusted for: age, gender, body mass index, smoking, systolic blood pressure, fasting plasma glucose, HDL-cholesterol, e-GFR, hs-CRP, and blood pressure target achievement during pharmacological treatment (yes/no). COPD: chronic obstructive pulmonary disease; e-GFR: estimated glomerular filtration rate; hs-CRP: high sensitivity C reactive protein; MI: myocardial infarction.

See the “Statistical analysis” section for all the information about the tests performed.

## Discussion

The major finding obtained in this observational prospective study conducted in a very large cohort of hypertensive patients is that COPD presence significantly increases the already established risk conferred by hypertension alone of incident stroke (fatal and non-fatal), MI (fatal and non-fatal) and MACE, regardless of other well established risk factors, during an average follow-up period of 57 months. The present study is, to our knowledge, the first that highlighted the adverse prognostic impact of COPD in addition to hypertension on the appearance of fatal and non-fatal cerebro-cardiovascular events. These findings consent to better stratify the risk of hypertensive patients because the coexistence of COPD encompasses the cardiovascular risk related to hypertensive status.

Essential HT remains the most predominant and modifiable risk factor for stroke, while only few studies investigated the relationship between COPD and stroke^12–14^. In particular, the Rotterdam Study^15^, a large prospective population-based cohort study, demonstrated that COPD increases the risk of stroke of about 20%, and this association was no longer significant after adjusting for smoking and other CV risk factors, such as HT. However, it is necessary to underline that our population is not comparable with that of the Rotterdam Study for age, smoking status, BP values and pharmacological treatment. The incidence of stroke in our cohort of hypertensives without COPD is concordant with previously published data obtained in the same setting of patients^16^. Additionally, and clinically relevant, results of our study show that, even after adjustment for BP values, the addition of COPD to hypertension increases of about nine- and 11-fold the risk of total and non-fatal stroke, respectively, despite the same percentage of patients who reached BP target. The clinical impact of these data grows from the fact that our COPD patients were classified as GOLD stage 1–2, condition often underdiagnosed and underestimated in clinical practice.

Similarly, the presence of COPD in hypertensives increases of about two-fold the risk of MI contributing to the global cardiovascular risk burden. Thus, on the basis of these findings, it is necessary to routinely investigate the presence of COPD in hypertensive patients, aiming to a very early diagnosis and treatment.

The potential pathophysiologic mechanisms linking COPD to an increased risk of CV events in HT can be identified in the subclinical chronic systemic inflammation^17^, typical of this disease, to the habit of cigarette smoking^18^ that is, per se, and independent CV risk factor for both coronary and cerebrovascular events, and to a dysregulation of the opioid system^19,20^. Both conditions are associated with an increase in oxidative stress and a decrease in the bioavailability of nitric oxide promoting coronary and extra-coronary vascular damage^21^. Moreover, abnormal lung function, including hyperinflation and hypoxia^22^, may have a negative impact on the CV system through several mechanisms: increased pressure in the cardiopulmonary system, right-ventricular dysfunction, impaired left-ventricular filling and reduced cardiac output^23^. In addition, in COPD patients coexist other clinical conditions such as lipid profile modifications, characterized by the increase of LDL-cholesterol oxidation and decrease of HDL-cholesterol^24^, and renal dysfunction^25^, that is an independent and strong predictor of CV morbidity and mortality^26,27^.

Furthermore, it is well established that COPD is an independent and strong predictor of incident atrial fibrillation^28^, which may be triggered by several insults such as pulmonary and hemodynamic abnormalities and consequent structural and functional modifications of right cardiac chambers, hypoxia and/or hypercapnia, electrophysiological alterations of the right atrium. Thus, the coexistence of COPD and atrial fibrillation can further increase the risk of both stroke and MI; for this survival analysis, we excluded patients who developed atrial fibrillation during the follow-up period in order to exclusively evaluate the effect of the coexistence of COPD and HT on the appearance of CV events, ruling out the potential confounding role of an increased pro-thrombotic status given by the concomitant presence of atrial fibrillation.

Notably, it is important to remark that our hypertensive patients with COPD, in comparison with hypertensive group without COPD, showed a significant reduction of e-GFR (81 vs 109 ml/min/1.73 m^2^) that in multivariable Cox regression analysis was retained as the second independent predictor of both fatal and non-fatal coronary outcomes. This finding has notable relevance in clinical practice because it is well established that renal function loss, as well as albuminuria^29,30^, are associated with an increased occurrence of both coronary and cerebrovascular events. Also in this case, the pathogenetic link is identifiable in the increased oxidative stress and endothelial dysfunction^31^, secondary to the elevation of asymmetric dimethylarginine (ADMA)^32^, a competitive antagonist of endothelial nitric oxide synthase. It is relevant to emphasize that ADMA is also associated with insulin resistance status^33^ and vascular aging^34^ that participate to the development of clinical outcomes. Thus, the clinical importance of our data come out from the fact that the negative impact exerted by COPD in hypertensive patients is independent of BP values and antihypertensive treatment; notably, COPD presence has a predominant impact on both fatal and non-fatal stroke occurrence rather than on coronary outcomes.

This study has several limitations: first of all the observational design does not allow to draw conclusions about a cause-effect relationship between COPD and the appearance of MACE; moreover, our results were obtained in hypertensive patients with GOLD stage 1–2 COPD, thus further studies are needed to confirm these findings in subjects in different GOLD stages.

In conclusion, our data demonstrate that COPD presence in hypertensive patients significantly increases both fatal and non-fatal stroke and total cardiovascular events occurrence.

## Methods

### Study population

**Figure 2:**
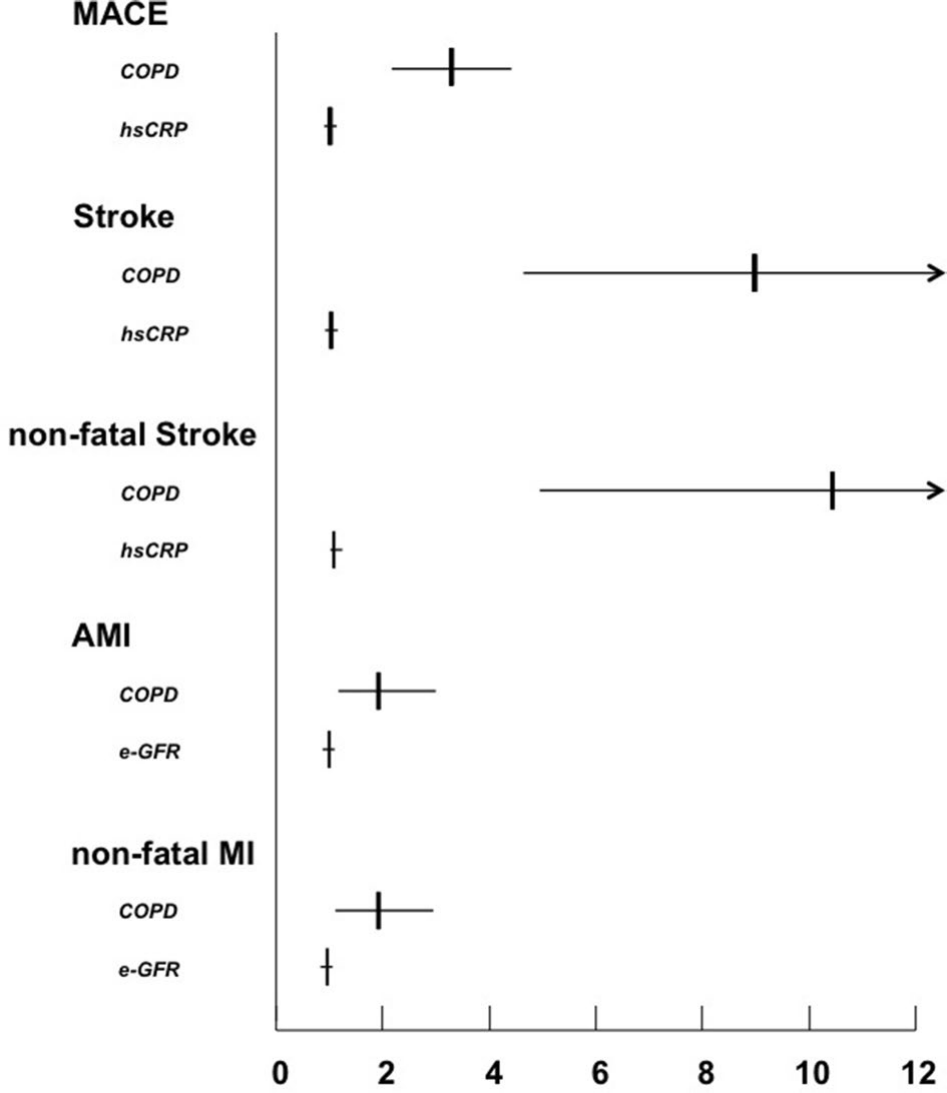
Flow chart of patients enrollment.

From an initial cohort 2195 of patients with essential HT, diagnosed according to the European Society of Cardiology guidelines^35^, and participating to the CATAnzaro MEtabolic RIsk factors (CATAMERI) Study^36^, we selected and prospectively followed over the time 1728 consecutive subjects referring to our Clinic Center, divided into two groups according with the presence/absence of COPD (Fig. 2). According with the aim of the study and to avoid possible interfering factors, patients developing atrial fibrillation during the follow-up were excluded from the analysis.

At the first evaluation, none of the patients had history or clinical evidence of cerebro- cardiovascular disease. Exclusion criteria were: diabetes mellitus defined as HbA1c ≥ 6.5% or fasting plasma glucose ≥ 126 mg/dl, chronic kidney disease (e-GFR < 60 ml/min/1.73  m^2^), liver and peripheral vascular disease, previous history or actual evidence of hypercoagulable syndrome or thrombophilia, hypercholesterolemia (total cholesterol > 200 mg/dl) and heart failure diagnosed according to both clinical and echocardiographic criteria. Secondary forms of hypertension were excluded by means of a standard clinical protocol, including renal ultrasound studies, computed tomography, renal scan, catecholamine, plasma renin activity, and aldosterone measurements.

The local Ethic Committee (Comitato Etico Calabria Centro) approved the study, and all participants gave written informed consent for all procedures according to the principles of the Declaration of Helsinki.

### Blood pressure measurements

According with current guidelines^35^, measurements of clinic BP were obtained in the left arm of seated patients, after 5 min of quiet rest, using a mercury sphygmomanometer, with a minimum of three BP readings on three different occasions at least 2 weeks apart. SBP and DBP were recorded at the first appearance and the disappearance of Korotkoff sounds. Baseline BP values were the average of the last two of the three consecutive measurements obtained at intervals of 3 min. Patients with a clinic SBP > 140 mmHg and/or DBP > 90 mmHg and/or use of antihypertensive drugs were defined as hypertensive.

### COPD assessment

Diagnosis of COPD was carried out according to medical history and by a standard spirometry performed by and trained and experienced personnel. Particularly, the established criterion for COPD were a post-bronchodilator forced expiratory volume in 1 s (FEV1)/forced vital capacity (FVC) ratio < 70%, and/or use of inhaled drugs, including long acting β2 adrenoceptor agonists (LABA) or/and muscarinic receptor antagonists (LAMA). Severity of COPD was defined according to Global Initiative for Chronic Obstructive Lung Disease (GOLD) criteria^37^, and in this analysis we included only patients with GOLD 1 and 2 stages because they represent the majority of our population (97%).

### Laboratory determinations

All laboratory measurements were performed after a fasting period of at least 12 h. Plasma glucose was determined by the glucose oxidase method (Glucose Analyzer, Beckman Coulter SpA, Milan, Italy). Triglyceride and total, low-density lipoprotein (LDL), and high-density lipoprotein (HDL) cholesterol concentrations were measured by enzymatic methods (Roche Diagnostics GmbH, Mannheim, Germany). Serum creatinine was measured by an automated technique based on the measurement of Jaffe chromogen and by the URICASE/POD method (Boehringer Mannheim, Mannheim, Germany) implemented in an auto-analyzer. Values of e-GFR were calculated by using the equation proposed by investigators in the Chronic Kidney Disease Epidemiology (CKD-EPI) Collaboration^38^. High-sensitivity C-reactive protein (hs-CRP) was measured by a turbidimetric immunoassay (Behring).

### Follow-up and cardiovascular events

All patients were treated to reduce clinic BP < 140/90 mmHg using standard lifestyle interventions and pharmacological treatment. Diuretics, β-blockers, ACE-inhibitors, calcium channel blockers (CCBs), angiotensin-II receptor blockers (ARBs), and α1-blockers were used alone or in various combinations. COPD therapy was administered according to GOLD recommendations (Table 2)^37^.

Follow-up included periodic clinical visits every six months or, if the patient was not able to perform it, a questionnaire was sent by mail to family physicians. All clinical events had to be validated by a local Committee on the basis of source data (hospital records, death certificates or other original documents). Fatal and non-fatal stroke, fatal and non-fatal MI, and other causes of CV death were recorded during the follow-up. For this analysis we considered the following: total CV events and CV deaths (MACE), total stroke and non-fatal stroke, total MI and non-fatal MI. MI diagnosis was defined according to criteria of the European Society of Cardiology/American College of Cardiology Foundation/American Heart Association/World Heart Federation^39^. Stroke was defined as a new neurological deficit of sudden onset that persisted for at least 24 h^40^. Other causes of CV deaths refers to CV deaths not included in the above categories but with a specific known cause (e.g., pulmonary embolism, malignant arrhythmia, end-stage heart failure, peripheral arterial disease, aortic rupture, etc.). Subjects free of CV events were defined as controls and the other patients were called cases.

### Statistical analysis

Data are summarized as mean and standard deviation (SD) (continuous variables) or as percentage (binary variables), and the comparison between two groups was made by independent *t*-test or Chi-Square test, as appropriate. For patients don't experiencing the study outcomes, the date of censor was that of the last contact. For patients who experienced multiple study outcomes, survival analysis was restricted to the first event. Kaplan–Meier curves were built for major adverse cardiac event (MACE), and both total MI and stroke occurrence. A log-rank test was performed to analyse differences in survival distributors between groups compared by using the Mantel (logistic rank) test. Univariate and multiple Cox regression analyses were performed to investigate the effect of COPD on the different study outcomes. In the univariate analyses we tested the following well recognized CV risk factors: age, gender, body mass index (BMI), smoking (current and former within 1 year), SBP, glucose, HDL-cholesterol, e-GFR, hs-CRP and BP target achievement during pharmacological treatment (yes/no). A multivariate survival model, adjusted for pharmacological treatment, was obtained by the Cox regression analysis including only variables significantly associated with study outcomes. In Cox models, data were expressed as hazard ratios (HR), 95% confidence intervals (CI) and P values. All calculations were done by SPSS for Windows Version 20 (Chicago, Illinois, USA).

## References

[1] World Mortality Report 2013. United Nations Department of Economic and Society Affairs, Population Division; 2013 http://www.un.org/en/development/desa/population/publications/pdf/mortality/WMR2013/World Mortality 2013. Report.pdf, Accessed 19 Sept. (2016).

[2] Violan C (2014). Prevalence, determinants and patterns of multimorbidity in primary care: A systematic review of observational studies. PLoS ONE.

[3] Yach D, Hawkes C, Gould CL, Hofman KJ (2004). The global burden of chronic diseases: Overcoming impediments to prevention and control. JAMA.

[4] Williams B, Mancia G, Spiering W, Agabiti Rosei E, Azizi M, Burnier M, Clement DL, Coca A, De Simone G, Dominiczak A, Kahan T (2018). The Task Force for the management of arterial hypertension of the European Society of Cardiology and the European Society of Hypertension. 2018 ESC/ESH Guidelines for the management of arterial hypertension. J. Hypertens..

[5] Fouad Rabahi M (2015). Prevalence of chronic obstructive pulmonary disease among patients with systemic arterial hypertension without respiratory symptoms. Int. J. Chron. Obstruct. Pulmon. Dis..

[6] Mullerova H, Agusti A, Ergon S, Marpel DW (2013). Cardiovascular comorbidity in COPD. Chest.

[7] Brown JP, Martinez CH (2016). Chronic obstructive pulmonary disease comorbidities. Curr. Opin. Pulm. Med..

[8] Trinkmann F, Saur J, Borggrefe M, Akin I (2019). Cardiovascular comorbidities in chronic obstructive pulmonary disease (COPD)—Current considerations for clinical practice. J. Clin. Med..

[9] Incalzi RA, Fuso L, Forastiere F, Rapiti E, Nardecchia B, Pistelli R (1997). Co-morbidity contributes to predict mortality of patients with chronic obstructive pulmonary disease. Eur. Respir. J..

[10] Zhang J, Rutten FH, Cramer MJ, Lammers JW, Zuithoff NP, Hoes AW (2011). The importance of cardiovascular disease for mortality in patients with COPD: A prognostic cohort study. Fam. Pract..

[11] Fumagalli G (2013). INDACO project: A pilot study on incidence of comorbidities in COPD patients referred to pneumology units. Multidiscip. Respir. Med..

[12] Feary JR, Rodrigues LC, Smith CJ, Hubbard RB, Gibson JE (2010). Prevalence of major comorbidities in subjects with COPD and incidence of myocardial infarction and stroke: A comprehensive analysis using data from primary care. Thorax.

[13] Finkelstein J, Cha E, Scharf SM (2009). Chronic obstructive pulmonary disease as an independent risk factor for cardiovascular morbidity. Int. J. Chron. Obstruct. Pulmon. Dis..

[14] Schneider C, Bothner U, Jick SS, Meier CR (2010). Chronic obstructive pulmonary disease and the risk of cardiovascular diseases. Eur. J. Epidemiol..

[15] Portegies ML (2016). Chronic obstructive pulmonary disease and the risk of stroke. The Rotterdam Study. Am. J. Respir. Crit. Care Med..

[16] Li C, Engström G, Hedblad B, Berglund G, Janzon L (2005). Blood pressure control and risk of stroke: A population-based prospective cohort study. Stroke.

[17] Fabbri LM, Rabe KF (2007). From COPD to chronic systemic inflammatory syndrome?. Lancet.

[18] Ambrose JA, Barua RS (2004). The pathophysiology of cigarette smoking and cardiovascular disease: An update. J. Am. Coll. Cardiol..

[19] Cozzolino D (2005). Acute pressor and hormonal effects of beta-endorphin at high doses in healthy and hypertensive subjects: Role of opioid receptor agonism. J. Clin. Endocrinol. Metab..

[20] Mahler DA (2009). Endogenous opioids modify dyspnoea during treadmill exercise in patients with COPD. Eur. Res. J..

[21] Perticone F (2001). Prognostic significance of endothelial dysfunction in hypertensive patients. Circulation.

[22] Torella D (2014). Carbonic anhydrase activation is associated with worsened pathological remodeling in human ischemic diabetic cardiomyopathy. J. Am. Heart Assoc..

[23] Rabe KF, Hurst JR, Suissa S (2018). Cardiovascular disease and COPD: Dangerous liasons?. Eur. Respir. Rev..

[24] Shen Y (2013). Increased serum ox-LDL levels correlated with lung function. Inflammation and oxidative stress in COPD. Mediators Inflamm..

[25] John M, Hussain S, Prayle A, Simms R, Cokcroft JR, Bolton CE (2013). Target renal damage: The microvascular association of increased aortic stiffness in patients with COPD. Respir. Res..

[26] Go AS, Chertow GM, Fan D, McCulloch CE, Hsu CY (2004). Chronic kidney disease and the risks of death, cardiovascular events, and hospitalization. N. Engl. J. Med..

[27] Fried LF (2003). Renal insufficiency as predictor of cardiovascular outcomes and mortality in elderly individuals. J. Am. Coll. Cardiol..

[28] Perticone M (2018). Competitive interaction between chronic obstructive pulmonary disease and CHA_2_DS_2_-VASc score in predicting incident atrial fibrillation. Int. J. Cardiol..

[29] Oelsner EC (2019). Albuminuria, lung function decline, and risk of incident chronic obstructive pulmonary disease. The NHLBI Pooled Cohorts Study. Am. J. Respir. Crit. Care Med..

[30] Meccariello A (2016). Microalbuminuria predicts the recurrence of cardiovascular events in patients with essential hypertension. J. Hpertens..

[31] Perticone F, Maio R, Tripepi G, Zoccali C (2004). Endothelial dysfunction and mild renal insufficiency in essential hypertension. Circulation.

[32] Perticone F (2005). Asymmetric dimethylarginine, l-arginine, and endothelial dysfunction in essential hypertension. J. Am. Coll. Cardiol..

[33] Perticone F (2010). Endothelial dysfunction, ADMA and insulin resistance in essential hypertension. Int. J. Cardiol..

[34] Scalera F, Martens-Lobenhoffer J, Bukowska A, Lendeckel U, Täger M, Bode-Böger SM (2008). Effects of telmisartan on nitric oxide-asymmetrical dimethylarginine system: Role of angiotensin II type 1 receptor gamma and peroxisome proliferator activated receptor gamma signaling during endothelial aging. Hypertension.

[35] Mancia, G. *et al.* The Task Force for the management of arterial hypertension of the European Society of Hypertension (ESH) and of the European Society of Cardiology (ESC). 2013 ESH/ESC Guidelines for the management of arterial hypertension. *J. Hypertens*. **31**, 1281–1357 (2013). 10.1097/01.hjh.0000431740.32696.cc 10.1097/01.hjh.0000431740.32696.cc 23817082

[36] Sesti G (2005). Plasma concentration of IGF-I is independently associated with insulin sensitivity in subjects with different degrees of glucose tolerance. Diabetes Care.

[37] GOLD. Global Strategy for the Diagnosis, Management and Prevention of COPD, Global Initiative for Chronic Obstructive Lung Disease (GOLD) 2017.

[38] Levey AS (2009). CKD-EPI (Chronic Kidney Disease Epidemiology Collaboration). A new equation to estimate glomerular filtration rate. Ann. Intern. Med..

[39] Thygesen K, Alpert JS, White HD, Jaffe AS, Apple FS, Galvani M, Katus HA, Newby LK, Ravkilde J, Chaitman B, Clemmensen PM, Dellborg M, Hod H, Porela P, Underwood R, Bax JJ, Beller GA, Bonow R, Van der Wall EE, Bassand JP, Wijns W, Ferguson TB, Steg PG, Uretsky BF, Williams DO, Armstrong PW, Antman EM, Fox KA, Hamm CW, Ohman EM, Simoons ML, Poole-Wilson PA, Gurfinkel EP, Lopez-Sendon JL, Pais P, Mendis S, Zhu JR, Wallentin LC, Fernández-Avilés F, Fox KM, Parkhomenko AN, Priori SG, Tendera M, Voipio-Pulkki LM, Vahanian A, Camm AJ, De Caterina R, Dean V, Dickstein K, Filippatos G, Funck-Brentano C, Hellemans I, Kristensen SD, McGregor K, Sechtem U, Silber S, Tendera M, Widimsky P, Zamorano JL, Morais J, Brener S, Harrington R, Morrow D, Lim M, Martinez-Rios MA, Steinhubl S, Levine GN, Gibler WB, Goff D, Tubaro M, Dudek D, Al-Attar N, Joint ESC/ACCF/AHA/WHF Task Force for the Redefinition of Myocardial Infarction (2007). Universal definition of myocardial infarction. Circulation.

[40] Adams HP (2007). Guidelines for the early management of adults with ischemic stroke: A guideline from the American Heart Association/American Stroke Association Stroke Council, Clinical Cardiology Council, Cardiovascular Radiology and Intervention Council, and the atherosclerotic peripheral vascular disease and quality of care outcomes in research interdisciplinary working groups: The American Academy of Neurology affirms the value of this guideline as an educational tool for neurologists. Circulation.

